# Supplementary material for: Global Archaeal Diversity Revealed Through Massive Data Integration: Uncovering Just Tip of Iceberg
Source: Microorganisms. 2025 Mar 5;13(3):598. doi: 10.3390/microorganisms13030598 (PMC11944491; doi:10.3390/microorganisms13030598)
Supplement: Supplementary file 1 [file microorganisms-13-00598-s001.zip › microorganisms-3470973-supplementary.pdf]

Supplement

March 4, 2025

Variable Region	Start	End	SINA start	SINA end
V1	8	96	1020	1789
V2	97	306	1791	6117
V3	307	487	6119	10289
V4	488	746	**10291**	**22460**
V5	747	885	**22461**	**27163**
V6	886	1029	27164	32813
V7	1030	1180	32814	37808
V8	1181	1372	37810	41570
V9	1373	1468	41572	43055

Table S1: Positioning of the nine variable and hyper-variable regions on the 16S rRNA gene of *Escherichia Coli* (strain K12) reference strain. Positions refer to both the whole gene and its aligned form with SINA. In bold the regions in the SINA alignment for the V4 and V5 regions that were used in our analysis.

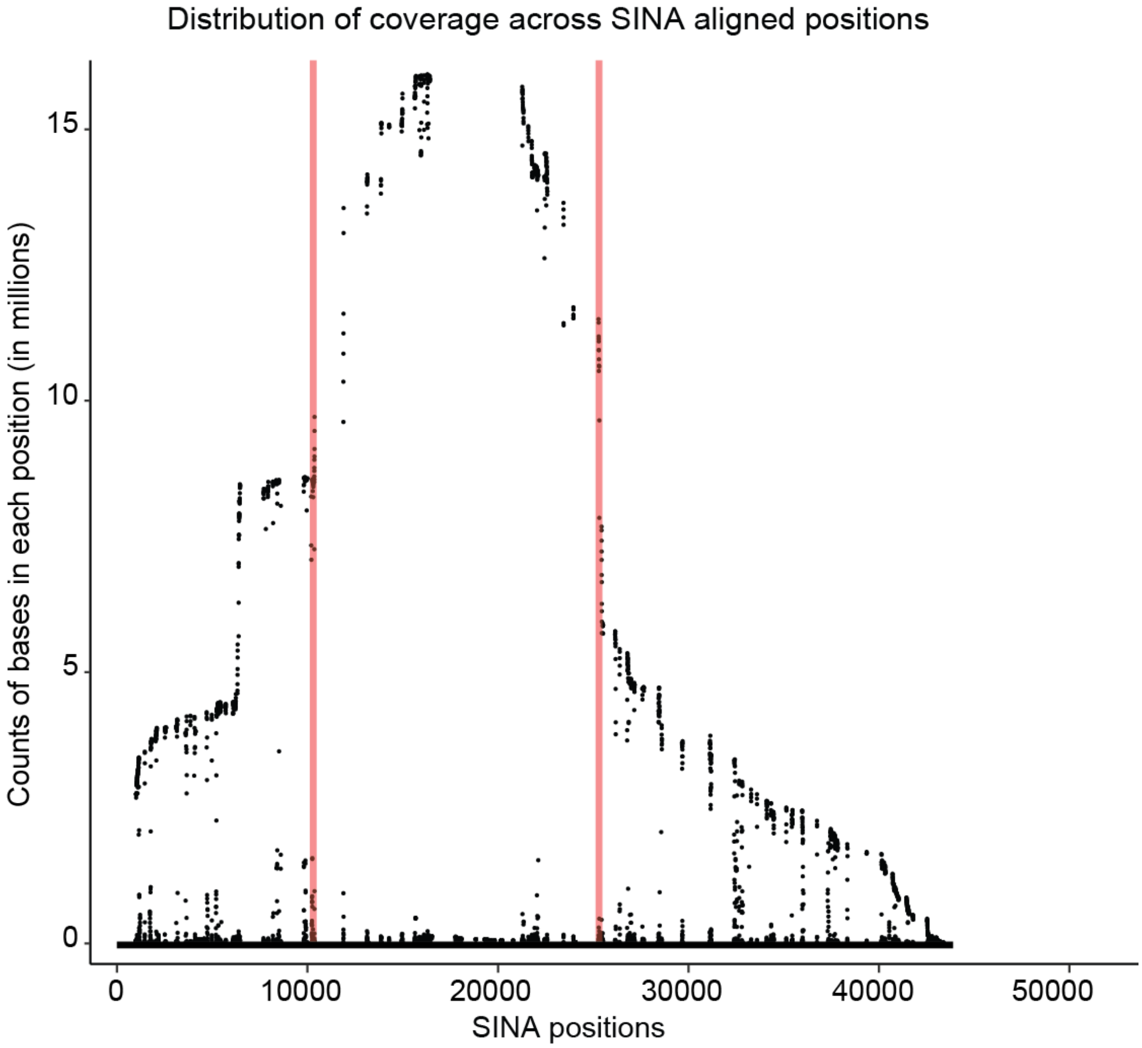

Figure S1: The archaeal Operational Taxonomic Units (OTUs) obtained from the IMNGS dataset were aligned using the SINA alignment tool. The figure illustrates the total number of aligned bases at each position across the alignment. The highlighted region corresponds to the specific segment referenced in the main text, representing a region that encapsulates the observed diversity and facilitates meaningful comparisons between samples.

Metagenome Origin	Count
gut metagenome	5692
bovine gut metagenome	2628
human gut metagenome	2315
Homo sapiens	992
feces metagenome	728
human metagenome	683
pig gut metagenome	671
Gallus gallus	565
pig metagenome	400
skin metagenome	277
mouse gut metagenome	260
bovine metagenome	233
human nasopharyngeal metagenome	219
fish gut metagenome	198
fish metagenome	189
mouse metagenome	165
Sus scrofa	136
insect gut metagenome	133
Equus caballus	118
rat gut metagenome	118
chicken gut metagenome	105
frog metagenome	81
human oral metagenome	75
Mus musculus	68
Sus scrofa domesticus	67
Bos taurus	63
human lung metagenome	61
crustacean metagenome	53
respiratory tract metagenome	46
primate metagenome	45
mosquito metagenome	45
insect metagenome	42
eye metagenome	37
human skin metagenome	37
Anas platyrhynchos	36
lung metagenome	28
beetle metagenome	27
Rattus norvegicus	26
oral metagenome	26
vaginal metagenome	24
human vaginal metagenome	24
upper respiratory tract metagenome	23
stomach metagenome	19
termite gut metagenome	18

Table S2: Table with the count of samples for each category of sample origin, as available in SRA, that indicate an animal host as a source. Due to poor annotation they were grouped as Host-Associated in this analysis.

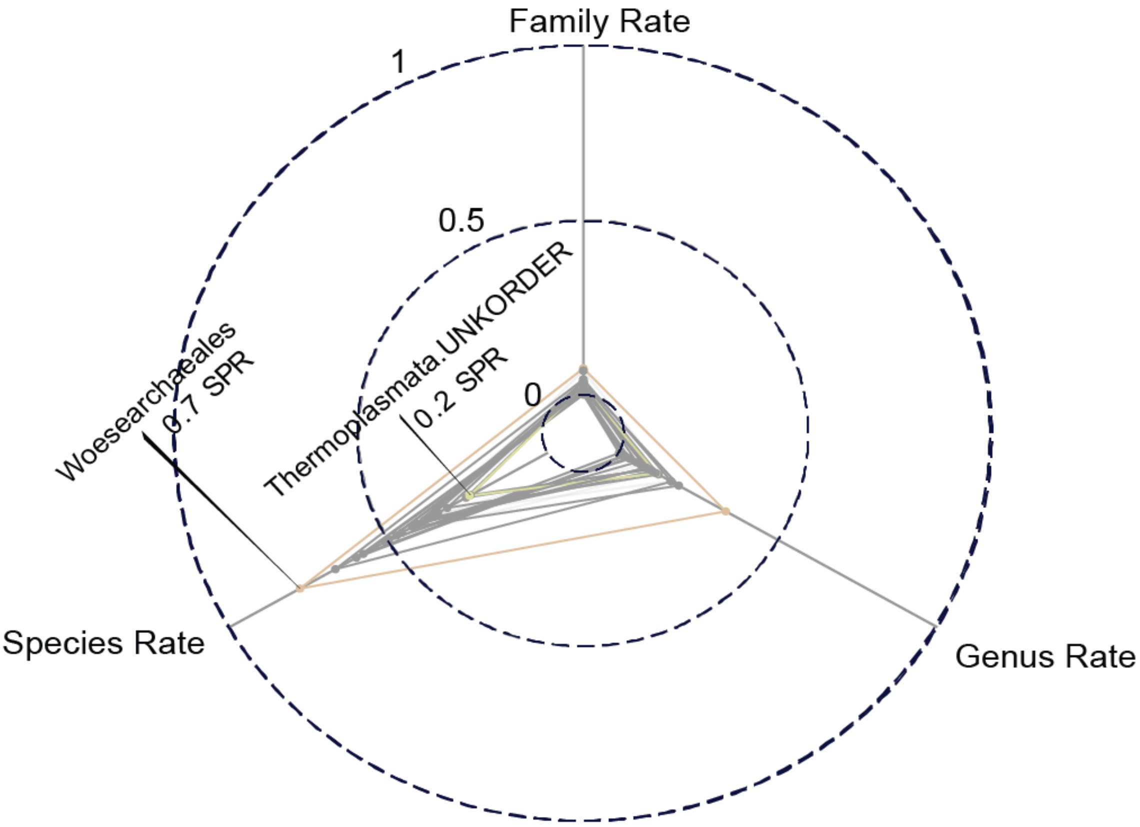

Figure S2: There are differences in the species and genera expansion rate among orders, with a minimum of 0.2 in the *Woesearcheales* order and a maximum of 0.7 in the unknown order contained within the class *Thermoplasmata*. The family expansion rate was uniform and constrained within rates of $[5 * 10^{-5}, 7 * 10^{-2}]$.

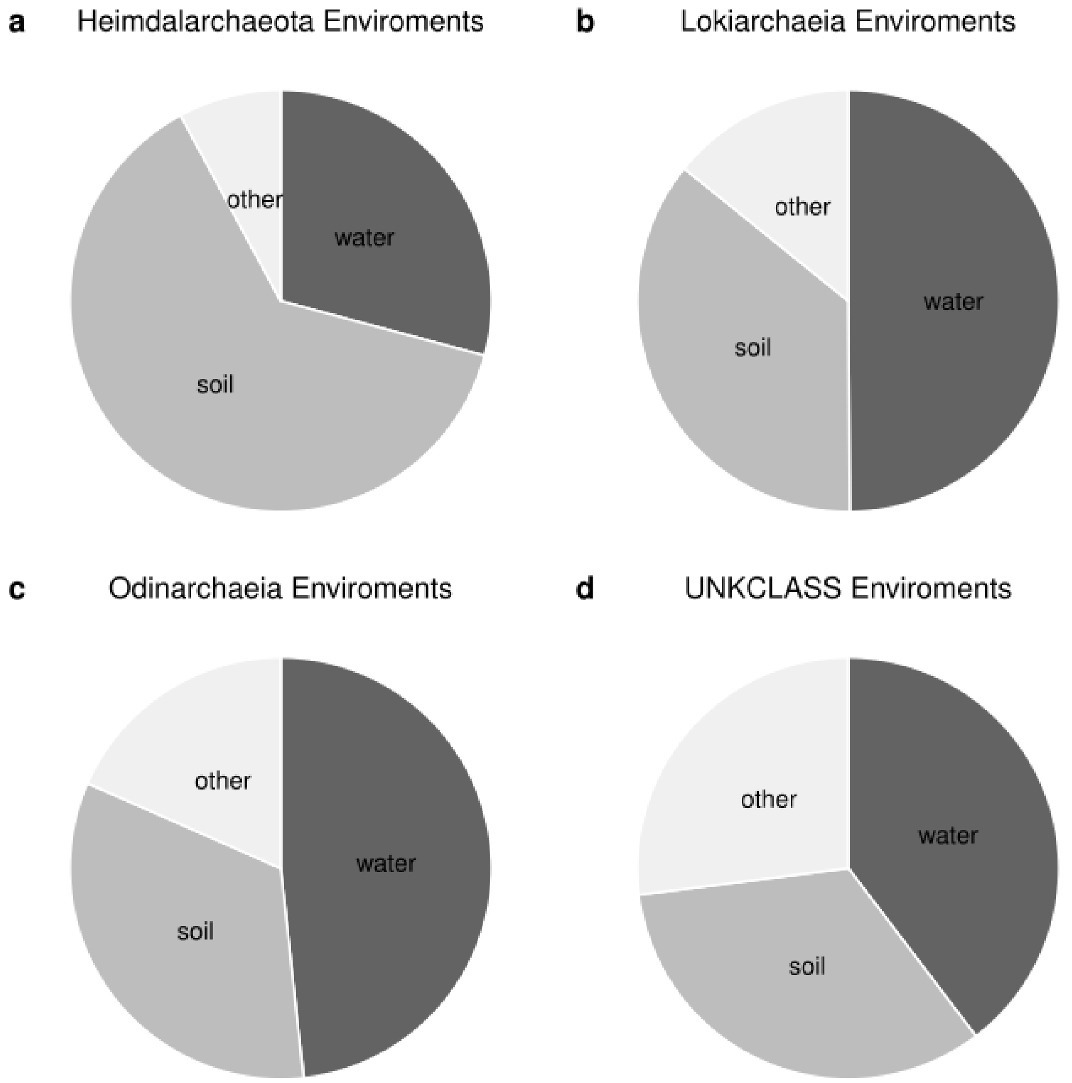

Figure S3: Enviromental preferences of *Asgardarcheota* classes.
